# Supplementary material for: A UMLS-based spell checker for natural language processing in vaccine safety
Source: BMC Med Inform Decis Mak. 2007 Feb 12;7:3. doi: 10.1186/1472-6947-7-3 (PMC1805499; doi:10.1186/1472-6947-7-3)
Supplement: Additional file 13 — RAP application directory. Sets up directory for RAP (RDF application for PHP) source code [file 1472-6947-7-3-S13.gz › rap/doc/phpdoc/__filesource/fsource_infModel__infModelInfModel.php.html]

xml version="1.0" encoding="iso-8859-1"?


File Source for InfModel.php


# Source for file InfModel.php

Documentation is available at InfModel.php

```
1. <?php
2. // ----------------------------------------------------------------------------------
3. // Class: infModel
4. // ----------------------------------------------------------------------------------

6. /**
7. * Class: InfModel
8. * @package infModel
9. */

11. /**
12. * A InfModel Model extends a MemModel , by adding the ability to infer statements from
13. * known statements and RDFS/OWL-Schematas.
14. * It uses the same interface as MemModel, thus making the
15. * infererence process hidden.
16. * <BR><BR>History:<UL>
17. * <LI>09-23-2004                 : Bug removed in _findRuleEntailmentInIndex().</LI>
18. * <LI>09-15-2004                 : Added Index over InRule Triggers
19. *                                   and Entailments to increase performance.</LI>
20. * <LI>09-10-2004                : First version of this class.</LI>
21. * </UL>
22. * @version  V0.9.1
23. * @author Daniel Westphal <mail at d-westphal dot de>
24. *
25. * @package infModel
26. * @access    public
27. */

29. class InfModel extends MemModel
30. {
31. /**
32. * array that holds the objects of the class Infrule,
33. * which were assigned by the _addToInference() function
34. * @var        array
35. * @access    private
36. */
37. var $infRules;

39. /**
40. * array of URI-Strings that produces Infrules
41. * @var        array
42. * @access    private
43. */
44. var $supportedInference;

46. /**
47. * array of the connection between the infrules and the statement
48. * that assigned those rules.
49. * array[2][3]=true;array[2][5]=true means, that statement 2
50. * assigned rule 3 & 5 to the model.
51. * @var        array
52. * @access    private
53. */
54. var $statementRuleIndex;

56. /**
57. * array of the infRule triggers and the matching infrules.
58. * $this->infRulesTriggerIndex['s'] for subject index, ['p'] for predicates,
59. * and ['o'] for objects.
60. * @var        array
61. * @access    private
62. */
63. var $infRulesTriggerIndex;

65. /**
66. * array of the infRule entailments and the matching infrules.
67. * $this->infRulesEntailIndex['s'] for subject index, ['p'] for predicates,
68. * and ['o'] for objects.
69. * @var        array
70. * @access    private
71. */
72. var $infRulesEntailIndex;


76. /**
77. * Constructor
78. * You can supply a base_uri
79. *
80. * @param string $baseURI
81. * @access    public
82. */
83. function InfModel ($baseURI = NULL)
84. {
85. //call the memmodel constructor method
86. parent::MemModel($baseURI);
87. //initialise vars
88. $this->infRulesTriggerIndex['s']=array();
89. $this->infRulesTriggerIndex['p']=array();
90. $this->infRulesTriggerIndex['o']=array();
91. $this->infRulesEntailIndex['s']=array();
92. $this->infRulesEntailIndex['p']=array();
93. $this->infRulesEntailIndex['o']=array();
94. $this->infRules=array();
95. $this->statementRuleIndex = array();
96. //arraylist of predicate labels that shall add inference rules
97. //to the model
98. //The constants, wich statements will create rules can be configured in constants.php
99. if (INF_RES_SUBCLASSOF)
100. $this->supportedInference[] = RDF_SCHEMA_URI.RDFS_SUBCLASSOF;

102. if (INF_RES_SUBPROPERTYOF)
103. $this->supportedInference[] = RDF_SCHEMA_URI.RDFS_SUBPROPERTYOF;

105. if (INF_RES_RANGE)
106. $this->supportedInference[] = RDF_SCHEMA_URI.RDFS_RANGE;

108. if (INF_RES_DOMAIN)
109. $this->supportedInference[] = RDF_SCHEMA_URI.RDFS_DOMAIN;

111. if (INF_RES_OWL_SAMEAS)
112. $this->supportedInference[] = OWL_URI.OWL_SAME_AS;

114. if (INF_RES_OWL_INVERSEOF)
115. $this->supportedInference[] = OWL_URI.OWL_INVERSE_OF;

117. //Rule: rdfs12
118. if (INF_RES_RULE_RDFS12)
119. {
120. $infRule=new InfRule();
121. $infRule->setTrigger(null,new Resource(RDF_NAMESPACE_URI.RDF_TYPE),new Resource(RDF_SCHEMA_URI.'ContainerMembershipProperty'));
122. $infRule->setEntailment('<s>',new Resource(RDF_SCHEMA_URI.RDFS_SUBPROPERTYOF),new Resource(RDF_SCHEMA_URI.'member'));
123. $this->_addInfRule($infRule,'base');
124. }

126. //Rule: rdfs6
127. if (INF_RES_RULE_RDFS6)
128. {
129. $infRule=new InfRule();
130. $infRule->setTrigger(null,new Resource(RDF_NAMESPACE_URI.RDF_TYPE),new Resource(RDF_NAMESPACE_URI.RDF_PROPERTY));
131. $infRule->setEntailment('<s>',new Resource(RDF_SCHEMA_URI.RDFS_SUBPROPERTYOF),'<s>');
132. $this->_addInfRule($infRule,'base');
133. }

135. //Rule: rdfs8
136. if (INF_RES_RULE_RDFS8)
137. {
138. $infRule=new InfRule();
139. $infRule->setTrigger(null,new Resource(RDF_NAMESPACE_URI.RDF_TYPE),new Resource(RDF_SCHEMA_URI.RDFS_CLASS));
140. $infRule->setEntailment('<s>',new Resource(RDF_SCHEMA_URI.RDFS_SUBCLASSOF),new Resource(RDF_SCHEMA_URI.RDFS_RESOURCE));
141. $this->_addInfRule($infRule,'base');

143. }

145. //Rule: rdfs10
146. if (INF_RES_RULE_RDFS10)
147. {
148. $infRule=new InfRule();
149. $infRule->setTrigger(null,new Resource(RDF_NAMESPACE_URI.RDF_TYPE),new Resource(RDF_SCHEMA_URI.RDFS_CLASS));
150. $infRule->setEntailment('<s>',new Resource(RDF_SCHEMA_URI.RDFS_SUBCLASSOF),'<s>');
151. $this->_addInfRule($infRule,'base');
152. }

154. //Rule: rdfs13
155. if (INF_RES_RULE_RDFS13)
156. {
157. $infRule=new InfRule();
158. $infRule->setTrigger(null,new Resource(RDF_NAMESPACE_URI.RDF_TYPE),new Resource(RDF_SCHEMA_URI.RDFS_DATATYPE));
159. $infRule->setEntailment('<s>',new Resource(RDF_SCHEMA_URI.RDFS_SUBCLASSOF),new Resource(RDF_SCHEMA_URI.RDFS_LITERAL));
160. $this->_addInfRule($infRule,'base');
161. }

163. }

165. /**
166. * Adds a new triple to the Model without checking if the statement
167. * is already in the Model.
168. * So if you want a duplicate free MemModel use the addWithoutDuplicates()
169. * function (which is slower then add())
170. * If the statement's predicate label is supported by the inference,
171. * the matching rules are added.
172. *
173. * @param    object Statement    $statement
174. * @access    public
175. * @throws    PhpError
176. */
177. function add($statement)
178. {
179. parent::add($statement);
180. //if the predicate is supported by the inference
181. if (in_array($statement->getLabelPredicate(),$this->supportedInference))
182. {
183. $this->_addToInference($statement);
184. };
185. }

187. /**
188. * This function analyses the statement's predicate and adds the
189. * matching infrule to the model
190. *
191. * @param    object Statement    $statement
192. * @access    private
193. */
194. function _addToInference($statement)
195. {
196. $predicateLabel=$statement->getLabelPredicate();
197. //get the position of the the statement in the model
198. end($this->triples);
199. $statementPosition=key($this->triples);

201. switch ($predicateLabel)
202. {
203. case RDF_SCHEMA_URI.RDFS_SUBPROPERTYOF :
204. //create a new rule
205. $infRule=new InfRule();
206. //set the trigger to match all statements, having a
207. //predicate, that matches the subject of the statement that
208. //created this rule.
209. $infRule->setTrigger(null,$statement->getSubject(),null);
210. //set the infrule to return a statement, having the same
211. //subject and object as the statement, that asked for an
212. //entailment, and having the object of the statement,
213. //that created this rule as predicate.
214. $infRule->setEntailment('<s>',$statement->getObject(),'<o>');
215. //add the infule to Model, Statement/Rule-Index,
216. //and Rule/Trigger (or Rule/Entailment) index
217. $this->_addInfRule($infRule,$statementPosition);
218. break;

220. case RDF_SCHEMA_URI.RDFS_SUBCLASSOF :
221. $infRule=new InfRule();
222. $infRule->setTrigger(null,new Resource(RDF_NAMESPACE_URI.RDF_TYPE),$statement->getSubject());
223. $infRule->setEntailment('<s>',new Resource(RDF_NAMESPACE_URI.RDF_TYPE),$statement->getObject());
224. $this->infRules[]=$infRule;
225. $this->_addInfRule($infRule,$statementPosition);
226. break;

228. case RDF_SCHEMA_URI.RDFS_DOMAIN :
229. $infRule=new InfRule();
230. $infRule->setTrigger(null,$statement->getSubject(),null);
231. $infRule->setEntailment('<s>',new Resource(RDF_NAMESPACE_URI.RDF_TYPE),$statement->getObject());
232. $this->infRules[]=$infRule;
233. $this->_addInfRule($infRule,$statementPosition);
234. break;

236. case RDF_SCHEMA_URI.RDFS_RANGE :
237. $infRule=new InfRule();
238. $infRule->setTrigger(null,$statement->getSubject(),null);
239. $infRule->setEntailment('<o>',new Resource(RDF_NAMESPACE_URI.RDF_TYPE),$statement->getObject());
240. $this->infRules[]=$infRule;
241. $this->_addInfRule($infRule,$statementPosition);
242. break;

244. case OWL_URI.OWL_INVERSE_OF :
245. $infRule=new InfRule();
246. $infRule->setTrigger(null,$statement->getSubject(),null);
247. $infRule->setEntailment('<o>',$statement->getObject(),'<s>');
248. $this->infRules[]=$infRule;
249. $this->_addInfRule($infRule,$statementPosition);

251. $infRule=new InfRule();
252. $infRule->setTrigger(null,$statement->getObject(),null);
253. $infRule->setEntailment('<o>',$statement->getSubject(),'<s>');
254. $this->infRules[]=$infRule;
255. $this->_addInfRule($infRule,$statementPosition);
256. break;

258. case OWL_URI.OWL_SAME_AS :
259. $infRule=new InfRule();
260. $infRule->setTrigger($statement->getSubject(),null,null);
261. $infRule->setEntailment($statement->getObject(),'<p>','<o>');
262. $this->_addInfRule($infRule,$statementPosition);

264. $infRule=new InfRule();
265. $infRule->setTrigger($statement->getObject(),null,null);
266. $infRule->setEntailment($statement->getSubject(),'<p>','<o>');
267. $this->_addInfRule($infRule,$statementPosition);

269. $infRule=new InfRule();
270. $infRule->setTrigger(null,$statement->getSubject(),null);
271. $infRule->setEntailment('<s>',$statement->getObject(),'<o>');
272. $this->_addInfRule($infRule,$statementPosition);

274. $infRule=new InfRule();
275. $infRule->setTrigger(null,$statement->getObject(),null);
276. $infRule->setEntailment('<s>',$statement->getSubject(),'<o>');
277. $this->_addInfRule($infRule,$statementPosition);

279. $infRule=new InfRule();
280. $infRule->setTrigger(null,null,$statement->getSubject());
281. $infRule->setEntailment('<s>','<p>',$statement->getObject());
282. $this->_addInfRule($infRule,$statementPosition);

284. $infRule=new InfRule();
285. $infRule->setTrigger(null,null,$statement->getObject());
286. $infRule->setEntailment('<s>','<p>',$statement->getSubject());
287. $this->_addInfRule($infRule,$statementPosition);
288. break;
289. };
290. }

292. /**
293. * This function checks, which infrules were added by the statement and
294. * removes those.
295. *
296. * @param    object Statement    $statement
297. * @return    integer
298. * @access    private
299. */
300. function _removeFromInference($statement)
301. {
302. $return= array();
303. $statementPosition=-1;
304. do
305. {
306. //get the position of the statement that should be removed
307. $statementPosition=$this->findFirstMatchOff($statement->getSubject(),
308. $statement->getPredicate(),
309. $statement->getObject(),
310. $statementPosition+1);
311. if ($statementPosition!=-1)
312. {
313. //if it added any rules
314. if (isset ($this->statementRuleIndex[$statementPosition]))
315. {
316. //remove all rules
317. foreach ($this->statementRuleIndex[$statementPosition] as $key => $value)
318. {
319. //remove from Rule-Trigger Index
320. if (is_a($this,'InfModelF'))
321. {
322. $trigger=$this->infRules[$key]->getTrigger();

324. if(is_a($trigger['s'],'Node'))
325. {
326. $subjectLabel=$trigger['s']->getLabel();
327. } else
328. {
329. $subjectLabel='null';
330. }
331. unset ($this->infRulesTriggerIndex['s'][$subjectLabel][array_search($key,$this->infRulesTriggerIndex['s'][$subjectLabel])]);

333. if(is_a($trigger['p'],'Node'))
334. {
335. $predicateLabel=$trigger['p']->getLabel();
336. } else
337. {
338. $predicateLabel='null';
339. }
340. unset ($this->infRulesTriggerIndex['p'][$predicateLabel][array_search($key,$this->infRulesTriggerIndex['p'][$predicateLabel])]);

342. if(is_a($trigger['o'],'Node'))
343. {
344. $objectLabel=$trigger['o']->getLabel();
345. } else
346. {
347. $objectLabel='null';
348. }
349. unset ($this->infRulesTriggerIndex['o'][$objectLabel][array_search($key,$this->infRulesTriggerIndex['o'][$objectLabel])]);
350. } else
351. //remove from Rule-Entailment Index
352. {
353. $entailment=$this->infRules[$key]->getEntailment();

355. if(is_a($entailment['s'],'Node'))
356. {
357. $subjectLabel=$entailment['s']->getLabel();
358. } else
359. {
360. $subjectLabel='null';
361. }
362. unset ($this->infRulesEntailIndex['s'][$subjectLabel][array_search($key,$this->infRulesEntailIndex['s'][$subjectLabel])]);


365. if(is_a($entailment['p'],'Node'))
366. {
367. $predicateLabel=$entailment['p']->getLabel();
368. } else
369. {
370. $predicateLabel='null';
371. }
372. unset ($this->infRulesEntailIndex['p'][$predicateLabel][array_search($key,$this->infRulesEntailIndex['p'][$predicateLabel])]);

374. if(is_a($entailment['o'],'Node'))
375. {
376. $objectLabel=$entailment['o']->getLabel();
377. } else
378. {
379. $objectLabel='null';
380. }
381. unset ($this->infRulesEntailIndex['o'][$objectLabel][array_search($key,$this->infRulesEntailIndex['o'][$objectLabel])]);
382. }
383. //remove from statement-Rule Index
384. unset ($this->infRules[$key]);
385. }
386. unset($this->statementRuleIndex[$statementPosition]);
387. $return[]=$statementPosition;
388. };
389. }

391. } while($statementPosition!=-1);

393. //return the positions of the statements to be removed OR emty array
394. //if nothing was found.
395. return $return;
396. }

398. /**
399. * Returns a model, containing all Statements, having a Predicate, that
400. * is supported by the inference.
401. *
402. * @return    object Model
403. * @access    public
404. */
405. function getSchema()
406. {
407. $res=new MemModel();
408. //Search the base-model for all statements, having a Predicate, that
409. //is supported by the inference.
410. foreach ($this->supportedInference as $inferencePredicateLabel)
411. {
412. $res->addModel($this->find(null, new Resource($inferencePredicateLabel), null));
413. }
414. return $res;
415. }

417. /**
418. * General method to replace nodes of a MemModel.
419. *
420. * This function is disabled in the Inference Model
421. *
422. * @param    object Node    $subject
423. * @param    object Node    $predicate
424. * @param    object Node    $object
425. * @param    object Node    $replacement
426. * @access    public
427. * @throws    PhpError
428. */
429. function replace($subject, $predicate, $object, $replacement)
430. {

432. $errmsg = RDFAPI_ERROR . '(class: InfModel; method: replace): This function is disabled in the Inference Model';
433. trigger_error($errmsg, E_USER_ERROR);
434. }

436. /**
437. * Method to search for triples using Perl-style regular expressions.
438. * NULL input for any parameter will match anything.
439. * Example:  $result = $m->find_regex( NULL, NULL, $regex );
440. * Finds all triples where the label of the object node matches the regular
441. * expression.
442. * Returns an empty MemModel if nothing is found.
443. *
444. * This function is disabled in the Inference Model
445. *
446. * @param    string    $subject_regex
447. * @param    string    $predicate_regex
448. * @param    string    $object_regex
449. * @return    object MemModel
450. * @access    public
451. */
452. function findRegex($subject_regex, $predicate_regex, $object_regex)
453. {

455. $errmsg = RDFAPI_ERROR . '(class: InfModel; method: findRegex):
456. This function is disabled in the
457. Inference Model';
458. trigger_error($errmsg, E_USER_ERROR);
459. }

461. /**
462. * Returns all tripels of a certain vocabulary.
463. * $vocabulary is the namespace of the vocabulary inluding a # : / char at
464. * the end.
465. * e.g. http://www.w3.org/2000/01/rdf-schema#
466. * Returns an empty MemModel if nothing is found.
467. *
468. * This function is disabled in the Inference Model
469. *
470. * @param    string    $vocabulary
471. * @return    object MemModel
472. * @access    public
473. */
474. function findVocabulary($vocabulary)
475. {

477. $errmsg = RDFAPI_ERROR . '(class: InfModel; method: findVocabulary):
478. This function is disabled in the
479. Inference Model';
480. trigger_error($errmsg, E_USER_ERROR);
481. }


484. /**
485. * Adds the URI or NULL to the Infrule trigger or entailment index
486. *
487. *
488. * @param    object infrule    $infRule
489. * @param    integer    $infRulePosition
490. * @access    private
491. */
492. function _addInfruleToIndex(& $infRule,& $infRulePosition)
493. {
494. //Add the rule only to the trigger index, if it is a InfFModel
495. if (is_a($this,'InfModelF'))
496. {
497. //get the trigger
498. $trigger = $infRule->getTrigger();
499. //evaluate and set the index
500. if ($trigger['s'] == null)
501. {
502. $this->infRulesTriggerIndex['s']['null'][]=$infRulePosition;
503. } else
504. {
505. $this->infRulesTriggerIndex['s'][$trigger['s']->getLabel()][]=$infRulePosition;
506. };

508. if ($trigger['p'] == null)
509. {
510. $this->infRulesTriggerIndex['p']['null'][]=$infRulePosition;
511. } else
512. {
513. $this->infRulesTriggerIndex['p'][$trigger['p']->getLabel()][]=$infRulePosition;
514. };

516. if ($trigger['o'] == null)
517. {
518. $this->infRulesTriggerIndex['o']['null'][]=$infRulePosition;
519. } else
520. {
521. $this->infRulesTriggerIndex['o'][$trigger['o']->getLabel()][]=$infRulePosition;
522. };
523. } else
524. //add to entailment Index if it is a BModel
525. {
526. //get the entailment
527. $entailment = $infRule->getEntailment();
528. //evaluate the entailment and add to index
529. if (!is_a($entailment['s'],'Node'))
530. {
531. $this->infRulesEntailIndex['s']['null'][]=$infRulePosition;
532. } else
533. {
534. $this->infRulesEntailIndex['s'][$entailment['s']->getLabel()][]=$infRulePosition;
535. };

537. if (!is_a($entailment['p'],'Node'))
538. {
539. $this->infRulesEntailIndex['p']['null'][]=$infRulePosition;
540. } else
541. {
542. $this->infRulesEntailIndex['p'][$entailment['p']->getLabel()][]=$infRulePosition;
543. };

545. if (!is_a($entailment['o'],'Node'))
546. {
547. $this->infRulesEntailIndex['o']['null'][]=$infRulePosition;
548. } else
549. {
550. $this->infRulesEntailIndex['o'][$entailment['o']->getLabel()][]=$infRulePosition;
551. };
552. };
553. }

555. /**
556. * Searches the trigger-index for a matching trigger and returns an
557. * array of infRule positions
558. *
559. *
560. * @param    object infrule    $infRule
561. * @return    array    integer
562. * @access    private
563. */
564. function _findRuleTriggerInIndex($statement)
565. {
566. $return=array();
567. //a statement's subject matches all triggers with null and the same URI
568. $subjectLabel=$statement->getLabelSubject();
569. $inIndexS=array();
570. if (isset($this->infRulesTriggerIndex['s']['null']))
571. $inIndexS=array_values($this->infRulesTriggerIndex['s']['null']);
572. if (isset($this->infRulesTriggerIndex['s'][$subjectLabel]))
573. $inIndexS= array_merge($subjectLabel,array_values($this->infRulesTriggerIndex['s'][$subjectLabel]));

575. //a statement's predicate matches all triggers with null and the same URI
576. $predicateLabel=$statement->getLabelPredicate();
577. $inIndexP=array();
578. if (isset($this->infRulesTriggerIndex['p']['null']))
579. $inIndexP=array_values($this->infRulesTriggerIndex['p']['null']);
580. if (isset($this->infRulesTriggerIndex['p'][$predicateLabel]))
581. $inIndexP= array_merge($inIndexP,array_values($this->infRulesTriggerIndex['p'][$predicateLabel]));

583. //a statement's object matches all triggers with null and the same URI
584. $objectLabel=$statement->getLabelObject();
585. $inIndexO=array();
586. if (isset($this->infRulesTriggerIndex['o']['null']))
587. $inIndexO=array_values($this->infRulesTriggerIndex['o']['null']);
588. if (isset($this->infRulesTriggerIndex['o'][$objectLabel]))
589. $inIndexO= array_merge($inIndexO,array_values($this->infRulesTriggerIndex['o'][$objectLabel]));

591. //if an infrule position occurs in subject, predicate, and object index, add to result array.
592. foreach ($inIndexP as $positionP)
593. {
594. if (in_array($positionP,$inIndexO))
595. if (in_array($positionP,$inIndexS))
596. $return[]=$positionP;
597. }
598. return $return;
599. }

601. /**
602. * Searches the Entailment-index for a matching Entailment and returns an
603. * array of infRule positions
604. *
605. *
606. * @param    node or null    $subject
607. * @param    node or null    $predicate
608. * @param    node or null    $object
609. * @return    array    integer
610. * @access    private
611. */
612. function _findRuleEntailmentInIndex($subject,$predicate,$object)
613. {
614. $return=array();
615. //a node matches all entailments with NULL or a matching URI
616. if(is_a($subject,'Node'))
617. {
618. $subjectLabel=$subject->getLabel();
619. $inIndexS=array();
620. if (isset($this->infRulesEntailIndex['s']['null'])) $inIndexS=array_values($this->infRulesEntailIndex['s']['null']);
621. if (isset($this->infRulesEntailIndex['s'][$subjectLabel])) $inIndexS= array_merge($inIndexS,array_values($this->infRulesEntailIndex['s'][$subjectLabel]));
622. } else
623. //if the subject search pattern is NULL, every rule will match the subject search patter
624. {
625. $inIndexS=array_keys($this->infRules);
626. }

628. if(is_a($predicate,'Node'))
629. {
630. $predicateLabel=$predicate->getLabel();
631. $inIndexP=array();
632. if (isset($this->infRulesEntailIndex['p']['null'])) $inIndexP=array_values($this->infRulesEntailIndex['p']['null']);
633. if (isset($this->infRulesEntailIndex['p'][$predicateLabel])) $inIndexP= array_merge($inIndexP,array_values($this->infRulesEntailIndex['p'][$predicateLabel]));
634. }  else
635. {
636. $inIndexP=array_keys($this->infRules);
637. }

639. if(is_a($object,'Node'))
640. {
641. $objectLabel=$object->getLabel();
642. $inIndexO=array();
643. if (isset($this->infRulesEntailIndex['o']['null'])) $inIndexO=array_values($this->infRulesEntailIndex['o']['null']);
644. if (isset($this->infRulesEntailIndex['o'][$objectLabel])) $inIndexO= array_merge($inIndexO,array_values($this->infRulesEntailIndex['o'][$objectLabel]));
645. } else
646. {
647. $inIndexO=array_keys($this->infRules);
648. }

650. //if an infrule position occurs in subject, predicate, and object index, add to result array.
651. foreach ($inIndexP as $positionP)
652. {
653. if (in_array($positionP,$inIndexO))
654. if (in_array($positionP,$inIndexS))
655. $return[]=$positionP;
656. }
657. return $return;
658. }

660. /**
661. * Adds an InfRule to the InfModel.
662. * $statementPosition states the positiion of the statement, that created
663. * this rule, in the model->triples array.
664. *
665. *
666. * @param    object Infrule    $infRule
667. * @param    integer    $statementPosition
668. * @access    private
669. */
670. function _addInfRule($infRule, $statementPosition)
671. {
672. //add the rule
673. $this->infRules[]=$infRule;
674. //get the position of the added rule in the model
675. end($this->infRules);
676. $rulePosition=key($this->infRules);
677. //add the information to the index, that this statement
678. //added this rule.
679. $this->statementRuleIndex[$statementPosition][$rulePosition]=true;
680. //add informations to index over trigger & entailment
681. $this->_addInfruleToIndex($infRule,$rulePosition);
682. }
683. }
684. ?>
```

Documentation generated on Thu, 7 Jul 2005 13:41:35 +0200 by phpDocumentor 1.3.0RC3
